# The Prevalence of Autoimmune Diseases Among Children With Type I Diabetes Mellitus

**DOI:** 10.7759/cureus.76272

**Published:** 2024-12-23

**Authors:** Eman S AlMoosa, Hussain A Al Ghadeer, Jumanah E Alatiya, Walaa H Aldairam, Ahmed M Alhamrani, Abdullah A Alarbash, Ali T Alamer, Manal T AlHelal, Abdullah M Alkhawajah, Zainab y AlDaif, Yaser A Alarab, Mohammed F Al Hani, Abdulelah Y Alhamdan, Akrm I AlWassel, Abdullah A Alshabaan

**Affiliations:** 1 Paediatrics, Maternity and Children Hospital, Al-Ahsa, SAU; 2 Paediatrics, Abqaiq General Hospital, Abqaiq , SAU; 3 Paediatrics, King Salman Hospital, Riyadh, SAU; 4 Paediatrics, Imam Abdulrahman Bin Faisal University, Dammam, SAU

**Keywords:** al-ahsa, autoimmune disease, prevalence, risk factors, saudi arabia, type 1 diabetes mellitus

## Abstract

Background

Type I diabetes mellitus (T1DM) is a prevalent chronic illness that typically manifests in childhood. In patients who are genetically predisposed to diabetes, complex interactions between environmental and genetic factors play a role in the development of type 1 diabetes. There is proof that the onset of type 1 diabetes raises the possibility of developing additional autoimmune conditions. This has to do with the hereditary predisposition to these illnesses. As the autoimmune process in pancreatic beta cells advances, it may also impact other organs, leading to the emergence of autoimmune diseases that are either organ-specific or organ-nonspecific.

Purpose

The purpose of this study is to determine the prevalence of autoimmune disorders among children who are diagnosed with T1DM in Al-Ahsa, Saudi Arabia, and assess the potential impact of these conditions on other comorbidities.

Methods

Over the course of three years, from 2020 to 2023, children with T1DM were the subjects of this descriptive retrospective cross-sectional study conducted in the Endocrinology and Diabetes Unit of the Maternity and Children's Hospital in Al-Ahsa, Saudi Arabia. There were 281 participants in total. Clinical and laboratory research was conducted on autoimmune T1DM.

Results

A total of 281 T1DM children were investigated, with 59.9% being female and 43.1% being male. The mean age was 12.8 ± 3.3 years, and the mean disease duration at the end of follow-up was 6.6 ± 2.9 years. Among these participants, 5.3% were diagnosed with at least one autoimmune disease (AID). Celiac disease is the most commonly reported AID, accounting for 56.3%, followed by hypothyroidism (31.3%). An increased risk of developing AIDs was linked to significant associations between older age (>10 years old) and longer duration of DM (p<0.05).

Conclusion

The data show a high prevalence of autoimmune comorbidities among pediatric T1DM patients treated at our department. The findings highlight the importance of regular screenings in facilitating timely diagnosis and intervention, which is critical in promoting the well-being and normative development of pediatric patients.

## Introduction

Type 1 diabetes mellitus (T1DM) is a chronic autoimmune disorder characterized by the destruction of beta cells in the pancreas, which leads to insulin deficiency and hyperglycemia [1]. T1DM is one of the most common chronic diseases in childhood, affecting approximately one in 500 children worldwide [2]. The presence of cellular immunity abnormalities, auto-antibodies against pancreatic islet cells, and infiltration of T-cells, B-cells, and macrophages by these cells have all demonstrated the autoimmune nature of T1DM. Both genetic and environmental factors have the potential to trigger the autoimmune process [3]. Moreover, autoimmunity can affect multiple organs and tissues, leading to non-organ-specific autoimmune diseases like rheumatoid arthritis, or it can affect other organs, resulting in organ-specific autoimmune diseases. Autoimmune thyroid disease, celiac disease, and autoimmune gastric disease are the three organ-specific autoimmune diseases most commonly linked to T1DM in children.

The co-occurrence of T1DM and autoimmune diseases (AIDs) in children has been shown to increase the risk of complications and poor glycemic control [4-7]. The prevalence of ADs among children with T1DM varies widely depending on the population studied and the methods used for diagnosis. Some studies have reported high rates of AIDs in children with T1DM, while others have found lower rates [8]. The identification of AIDs in children with T1DM is critical as it can affect the management and treatment of both conditions [9]. Therefore, a better understanding of the prevalence of AIDs in children with T1DM is essential for optimizing clinical care and improving outcomes in this vulnerable population. This research aims to investigate the prevalence and types of AIDs in children with T1DM in Al-Ahsa, Saudi Arabia. The study will explore the association between AIDs and T1DM and assess the impact of co-occurring AIDs on glycemic control and the risk of complications. The findings of this study will provide important insights into the burden of AIDs in children with T1DM and inform the development of personalized care plans for this population.

## Materials and methods

Aim of the study

The purpose of this study is to determine the prevalence of autoimmune disorders among children who are diagnosed with T1DM in Al-Ahsa and assess the potential impact of these conditions on other comorbidities.

Study design and population

This study was conducted in the Endocrinology and Diabetes Unit of the Maternity and Children's Hospital in Al-Ahsa. Between January 2020 and December 2023, computerized records for T1DM patients with and without autoimmune diseases were reviewed. All patient information was thoroughly documented in the patient files, resulting in a clear database of data that could be evaluated. Age, gender, anthropometric measurements (height, weight, body mass index (BMI)), and laboratory results were collected and analyzed for 281 individuals with T1DM with/without autoimmune diseases. Autoimmune disorders involve a wide spectrum of illnesses that can be diagnosed clinically or in a laboratory. Any patient less than 14 years old with T1DM and on regular follow-up were included in the study.

Data analysis

After data were extracted, they were revised, coded, and fed to statistical software using IBM SPSS Statistics, Version 22 (Released 2013; IBM Corp., Armonk, New York, United States). All statistical analysis was done using two-tailed tests. A p-value less than 0.05 was statistically significant. A descriptive analysis based on frequency and percent distribution was done for all variables, including children's bio-demographic data, personal and family history of AID, and diabetes-related data with vitamin D level that was graphed. Also, AID prevalence, types, and age at diagnosis were also tabulated. Cross tabulation was used to assess factors associated with AIDs among type II diabetic children using Person's chi-square test and exact probability test for small frequency distributions. The mean vitamin D level and glycated haemoglobin (HbA1c) were compared among children by their AID history using independent samples t-test.

## Results

A total of 281 type I diabetic children were included. Children's ages ranged from a few months to 18 years, with a mean age of 12.8 ± 3.3 years old. Exactly 160 (56.9%) children were females. Considering BMI, 110 (39.1%) were underweight, 32 (11.4%) were overweight, and nine (3.2%) were obese. As for chronic diseases, 22 (7.8%) had glucose-6-phosphate dehydrogenase (G6PD) deficiency, eight (2.8%) had hypothyroidism, and two (0.7%) had autism spectrum disorder (ASD), two (0.7%) had celiac disease, and two (0.7%) had sickle cell disease (SCD). The vast majority of the children (85.4%; 240) had no chronic health problem (Table 1).

**Table 1:** Bio-demographic characteristics of study children with type I DM (n=281)

Bio-demographic data	No	%
Age in years		
Toddler (1-5 years)	11	3.9%
School-aged (6-10 years)	43	15.3%
>10 years	227	80.8%
Mean SD	12.8 ± 3.3	
Gender		
Male	121	43.1%
Female	160	56.9%
Body mass index		
Underweight	110	39.1%
Normal weight	130	46.3%
Overweight	32	11.4%
Obese	9	3.2%
Chronic disease		
None	240	85.4%
Glucose-6-phosphate dehydrogenase (G6PD) deficiency	22	7.8%
Hypothyroidism	8	2.8%
Autism	3	1.1%
Celiac disease	2	.7%
Sickle cell disease (SCD)	2	.7%
Anxiety disorder	1	.4%
Bronchial asthma	1	.4%
Epilepsy	1	.4%
Mauriac Syndrome	1	.4%

Diabetes mellitus-related data was collected from study children in Al-Ahsa, Saudi Arabia (Table 2). A total of 129 (45.9%) children were diagnosed with type I DM at the age of one-to-five years, 110 (39.1%) were diagnosed during their six-to-10 years of age, and 42 (14.9%) were diagnosed at greater than 10 years of age. The duration of DM ranged from two to 16 years, with a mean duration of 6.6 ± 2.9 years. A total of 255 (90.7%) children were on a multiple daily injection (MDI) regimen, and only 26 (9.3%) were on Insulin Pump Therapy. As for DM-related complications, 21 (7.7%) had diabetic ketoacidosis (DKA) once, six (2.2%) had DKA twice, four (1.5%) had hypothyroidism, but most of the children (88.6%) had no complications. The HbA1C average level was 9.0 ± 1.8%. A total of 70 (24.9%) had uncontrolled HbA1C during the last year.

**Table 2:** Diabetes mellitus (DM)-related data among children

DM data	No	%
Age at diagnosis		
1-5	129	45.9%
6-10	110	39.1%
>10	42	14.9%
Duration of DM		
< 5 years	105	37.4%
5-10 years	137	48.8%
> 10 years	39	13.9%
Mean ± SD	6.6 ± 2.9	
Insulin regimen		
Multiple daily injection (MDI)	255	90.7%
Pump	26	9.3%
Complications during last year		
None	244	90.1%
Diabetic ketoacidosis (DKA) once	21	7.7%
Diabetic ketoacidosis (DKA) twice	6	2.2%
Hemoglobin A1C during the last year		
Controlled <7%	18	6.4%
Suboptimal 7-10%	193	68.7%
Uncontrolled >10%	70	24.9%
Mean ± SD	9.0 ± 1.8	

Prevalence, pattern, and family history of autoimmune diseases among type I DM children in Al-Ahsa, Saudi Arabia, are given in Table 3. Only 15 (5.3%) diabetic children had AID, mainly celiac disease (56.3%; nine), hypothyroidism (31.3%; five), alopecia (6.3%; one), and vitiligo (6.3%; one). The majority of autoimmune disorders, such as celiac disease (90%) and hypothyroidism (80%), were identified after the patient was diagnosed with DM. A total of 118 (42%) children had a family history of AID, mainly second-degree (54.2%), then first-degree (24.6%), and third-degree (21.2%).

**Table 3:** Prevalence, pattern, and family history of autoimmune diseases among type I DM children

Autoimmune diseases	No	%
Autoimmune disease		
Yes	15	5.3%
No	266	94.7%
Type of disease		
Celiac	9	56.3%
Hypothyroidism	5	31.3%
Alopecia	1	6.3%
Vitiligo	1	6.3%
Hypothyroidism time of diagnosis		
After DM	4	80.0%
Before DM	1	20.0%
Celiac disease time of diagnosis		
After DM	9	90.0%
With DM	1	10.0%
Alopecia time of diagnosis		
After DM	1	100.0%
Vitiligo time of diagnosis		
After DM	1	100.0%
Family history of autoimmune disease		
Yes	118	42.0%
No	163	58.0%
What is the degree of relatives?		
1st (parents)	23	19.5%
1st (siblings)	6	5.1%
2nd (aunts/uncles)	41	34.7%
2nd (grandparents)	23	19.5%
3rd	25	21.2%

**Figure 1:**
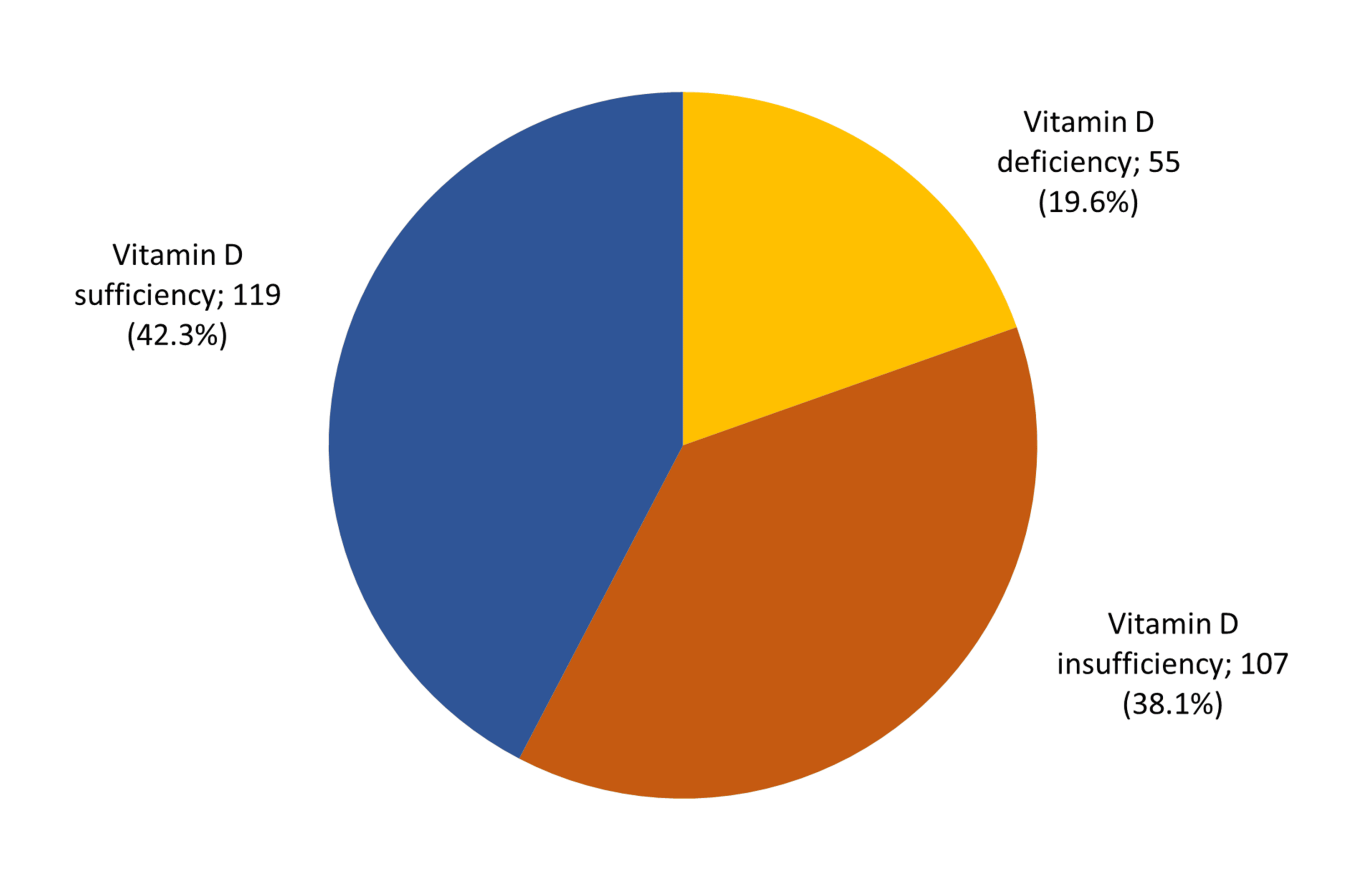
Vitamin D levels among children with type I DM children

Vitamin D levels among type 2 DM children in Al-Ahsa are given in Figure 1. A total of 55 (19.6%) had vitamin D deficiency, 107 (38.1%) had vitamin D insufficiency, and 119 (42.3%) had vitamin D sufficiency.

Factors associated with having autoimmune diseases among type I DM children in Al-Ahsa (Table 4). A total of 6.6% of children aged more than 10 years had AID versus none of the others with a recorded statistical significance (p-value=.049). Also, 12.8% of diabetic children with DM for more than 10 years had AIDs compared to 1% of others with DM for less than five years (p-value=.013).

**Table 4:** Factors associated with having autoimmune disease among children with type I DM children ^: Exact probability test; #: Independent t-test; * p-value < 0.05 (significant)

Factor	Autoimmune disease				p-value
	Yes		No		
	No	%	No	%	
Age in years					.049*^
Toddler (1-5 years)	0	0.0%	11	100.0%	
School-aged (6-10 years)	0	0.0%	43	100%	
> 10 years	15	6.6%	212	93.4%	
Gender					.409
Male	8	6.6%	113	93.4%	
Female	7	4.4%	153	95.6%	
Body mass index					.607^
Underweight	4	3.6%	106	96.4%	
Normal weight	9	6.9%	121	93.1%	
Overweight	2	6.3%	30	93.8%	
Obese	0	0.0%	9	100.0%	
Age at diagnosis					.511
1-5	9	7.0%	120	93.0%	
6-10	4	3.6%	106	96.4%	
>10	2	4.8%	40	95.2%	
Duration of DM					.013*
< 5 years	1	1.0%	104	99.0%	
5-10 years	9	6.6%	128	93.4%	
> 10 years	5	12.8%	34	87.2%	
Insulin regimen					.893^
Multiple daily injection (MDI)	14	5.5%	241	94.5%	
Pump	1	4.3%	25	95.7%	
Complications during last year					.813
None	13	5.4%	227	94.6%	
Diabetic ketoacidosis once	2	6.5%	29	93.5%	
Family history of autoimmune disease					.872
Yes	6	5.1%	112	94.9%	
No	9	5.5%	154	94.5%	
HbA1c					.900^#^
Mean ± SD	9.0 ± 3.2		9.0 ± 1.7		
Vitamin D					.377^#^
Mean ± SD	17.6 ± 9.2		19.7 ± 9.1		

## Discussion

The current study aimed to assess the prevalence of autoimmune diseases among children with type I DM in Al-Ahsa, Saudi Arabia. The onset of additional AIDs in the same individual during T1DM has been of interest, suggesting a strongly shared genetic susceptibility and shared pathological mechanisms with other AIDs since the autoimmune reaction may spread to the other systems, resulting in comorbidities [10]. The development of alternative autoantibodies linked to a distinct disease categorization within the set of a particular AID facilitates the transmission of autoimmunity across disease-specific borders [11]. This phenomenon, known as polyautoimmunity, has been proposed as a factor in the development and later clinical presentation of patients [12].

The current study revealed that most diabetic children aged more than 10 years are underweight or have normal body weight. Also, most of them had no other co-morbidity. Considering diabetes data, most of the children were diagnosed below the age of 10 years using the MDI regimen. The vast majority had suboptimal diabetes control with no related complications. DKA was the most reported diabetes-related complication.

With regard to the prevalence of AIDs, the current study revealed that only 5% of diabetic children had AIDS, mainly celiac disease and hypothyroidism. Most cases had AIDs after DM diagnosis. With regard to family history, less than half of the children had one family member with AIDs, mainly second- and first-degree relatives. Old age (more than 10 years) and long duration of DM were significantly associated with having AIDs. Similar to the current study, Grasso and Chiarelli [13] found that thyroid disorders and celiac disease are the most common AIDs diagnosed in children and adolescents with T1DM, but it's also critical to take into account the onset of other conditions like juvenile idiopathic arthritis, multiple sclerosis, atrophic gastritis, inflammatory bowel diseases, and skin conditions like vitiligo and psoriasis. The literature revealed that the global prevalence of AIDs in children is about 5%, with the most frequent ones being autoimmune thyroid diseases, which are consistent with the current study's findings [14,15]. In the United States, Maahs et al. [16] documented a prevalence of one case per 300 children. Nonetheless, research including 179 children with diabetes found that 2.8% of the cohort had another AID at the time of the T1DM diagnosis, and 15.6% of the group tested positive for additional autoantibodies (anti-transglutaminase, anti-thyroglobulin, or anti-thyroid peroxidase antibodies) [14].

Furthermore, throughout follow-up, the frequency of both antibody positive and the emergence of new AIDs increased gradually. The literature also revealed that about 4.6% to 16.4% of T1DM patients had coeliac disease (CD), and like T1DM, its incidence has gradually increased over the past 30 years, but it is now beginning to level off [17-21]. The increased likelihood of both illnesses arising is explained by shared genetic risk factors [22-24]. Such other AIDs, CD, and T1DM have been connected to prior infections as well as other environmental variables such as socioeconomic position, imbalanced gut flora, and insufficient vitamin D [20].

While the exact cause of the immunological cascade in T1DM is uncertain, dietary gluten has been found to be the causal antigen for CD. Also, it has been widely noted that there is a 15% to 30% relation between T1DM and thyroid disorders [25-28]. As a result, the American Academy of Diabetes advises screening for autoimmune thyroid disease both immediately following the diagnosis and then on a recurring basis [29]. Polyglandular syndrome type 2, which is commonly characterized by Addison's disease, autoimmune thyroid disease, and/or type 1 diabetes, also exhibits an overlap between these two disorders. Thus, at the onset of T1DM, once the child is clinically stable, National and International Guidelines for Diabetes Management recommend screening for anti-thyroid peroxidase and anti-transglutaminase antibodies, TSH, and free T4. Additionally, these patients should have their thyroid function monitored for the duration of their lives [30,31]. Twenty-five percent of children have positive thyroid antibodies at the start of diabetes. Thyroid peroxidase antibodies outperform anti-thyroglobulin antibodies in terms of predictiveness [30].

Patients with T1DM had a 2% to 10% prevalence of vitiligo [14]. Vitiligo was discovered in five out of a cohort of 300 young T1DM patients, representing 1.6% of the overall population, as opposed to 0.7% of the general population [32]. Studies that examined the function of CD-4 positive T-cells in both vitiligo and T1DM have addressed the connection between the two conditions [33,34], albeit a particular target shared by both disorders has not yet been identified.

Limitation

The patient data may not be typical of the entire region because it was only collected from one institution. A register-based study's results are entirely reliant on the quality of the registrations, with potential errors from inaccurate registrations posing restrictions. An extended follow-up period in a comparable research might also be intriguing. It is important to investigate the unknown risk of long-term consequences in people with T1DM and autoimmune comorbidity.

## Conclusions

The current study assessed a consistent prevalence of AIDs with the global assessed frequency, mainly celiac disease and hypothyroidism, which were significantly higher among old-aged diabetic children with long DM duration. Diabetic children with one or more AIDs need interdisciplinary care that is well-coordinated, and patients and their families require support. Assessing the frequency of these conditions, the genes implicated, the best time to screen, and customizing treatment for each patient require significant advancements. On the other hand, a more thorough comprehension of the similarities and distinctions among autoimmune diseases would hasten the process of information transfer toward an earlier diagnosis and more efficient, customized treatment.
